# The prevalence of an isolated gastrocnemius tightness in patients with knee or hip pathology

**DOI:** 10.3389/fresc.2026.1733301

**Published:** 2026-04-07

**Authors:** Špela Kralj Vitez, Matej Drobnič, Tina Tomc Žargi

**Affiliations:** 1 Faculty of Health Sciences, Department of Physiotherapy, University of Ljubljana, Ljubljana, Slovenia; 2 Department of Orthopaedic Surgery, University Medical Centre Ljubljana, Ljubljana, Slovenia; 3 Chair of Orthopaedics, Faculty of Medicine, University of Ljubljana, Ljubljana, Slovenia

**Keywords:** activity level, arthroplasty, arthroscopy, isolated gastrocnemius tightness, weight-bearing lunge test

## Abstract

**Introduction:**

Clinically, isolated gastrocnemius tightness (IGT) is identified by limited ankle dorsiflexion when the knee is extended, which improves with knee flexion. The Weight Bearing Lunge Test is the preferred method for assessing IGT due to its high reliability. While IGT is well-documented in foot pathology, its prevalence in knee and hip pathologies remains underexplored. The aim of this study was to determine the prevalence and extent of isolated gastrocnemius tightness before elective surgery in patients with knee or hip pathology and to confirm correlations between isolated gastrocnemius tightness and patients' demographics, pain, and activity levels.

**Methods:**

Eighty patients admitted to a tertiary orthopaedic centre for elective surgery (total arthroplasty or arthroscopy) were included. Demographics, pain, and activity levels were measured. Ankle dorsiflexion index was measured bilaterally with a Weight-Bearing Lunge Test by using a gravity inclinometer.

**Results:**

Gastrocnemius tightness was defined as an ankle dorsiflexion index ≥ 13°. The mean bilateral ankle dorsiflexion index was 8° ± 4°. Based on this criterion, isolated gastrocnemius tightness was identified in 14% of patients. When a less strict definition was applied (ankle dorsiflexion index ≥ 10°), the prevalence of isolated gastrocnemius tightness increased to 37% of patients. Total hip arthroplasty patients had significantly higher dorsiflexion index on both limbs (treated: 11° ± 5°, control; 10° ± 3°) than other groups (total knee arthroplasty 7° ± 3°; hip arthroscopy 7° ± 4°; knee arthroscopy 6° ± 3°). Gastrocnemius tightness was present in 21% of patients with hip pathology, compared to 8% in those with knee pathology. No significant differences were found between treated and control legs.

**Conclusions:**

Ankle dorsiflexion index correlated positively with age and pain but negatively with activity level. One in five patients with hip pathology and one in ten with knee pathology exhibited isolated gastrocnemius tightness. Total hip arthroplasty patients presented with significantly higher ankle dorsiflexion index than other groups. Increased age and pain levels were associated with increased gastrocnemius tightness, while a higher activity level appeared protective.

## 1 Introduction

The non-neuromuscular, non-paralytic form of isolated gastrocnemius tightness (IGT) has been increasingly recognized as a contributing factor in the development of forefoot and/or midfoot pathologies, even among otherwise healthy individuals (1–4). Amis (5) provided a comprehensive biomechanical explanation detailing how isolated shortening of the gastrocnemius muscle can lead to abnormal loading patterns and subsequent structural damage in the lower leg, ankle, and foot. In a study conducted by DiGiovanni et al. (6), the prevalence of IGT was found to be 24% in a generally healthy adult population, with a substantially higher prevalence of 65% among individuals presenting with various foot disorders. These findings are further supported by Malhotra et al. (7), who reported an exceptionally high prevalence of IGT—up to 97%—in patients specifically diagnosed with forefoot pathology.

In addition to its relationship with foot-related conditions, a possible association between IGT and lower back pain has also been observed, as reported (8), suggesting a broader impact of gastrocnemius tightness on musculoskeletal function beyond the distal lower limb. Despite these observations, the literature remains limited with respect to the prevalence and clinical relevance of IGT in relation to pathologies affecting more proximal joints of the lower extremity, such as the knee and hip. Consequently, further investigation is warranted to elucidate the role of IGT in the biomechanical context of these larger joints and its potential contribution to disease development or symptom exacerbation in specified regions.

Gastrocnemius is a triarticular muscle spanning the knee and ankle joints. As a result, gastrocnemius tightness limits ankle dorsiflexion with the knee extended, while knee flexion reduces its effect and increases dorsiflexion, forming the biomechanical basis of isolated gastrocnemius tightness (5). Clinically, IGT is manifested by limited passive dorsiflexion of the ankle performed with the extended knee, whereas knee bending increases or even normalises dorsiflexion of the ankle. If dorsiflexion is restricted through both parts of the test in the absence of ankle pathology, combined gastrocnemius-soleus shortening is most commonly the cause of range of motion restriction (1). Baumbach et al. (9) showed that 20° of knee flexion completely abolishes the restraining effect of the gastrocnemius on the ankle dorsiflexion. A schematic illustration of the anatomical and testing principles underlying the relationship between ankle dorsiflexion and knee position is presented in a previous study by Baumbach et al. (10). The most used clinical test to detect IGT is the Silfverskiöld test, but due to its low reliability, it has been discontinued for quantitative analyses (11). Recently, the Weight Bearing Lunge Test (WBLT) has been recommended for the investigational and clinical evaluation of IGT. The WBLT, as illustrated in a study by Baumbach et al. (9), is easily reproducible and has high to excellent reliability (12–15). Dickson et al. (16) proposed placing a gravity inclinometer directly over the tuber calcanei for optimal measurement results. This method was shown to have lower perceptible changes compared to a digital or a classical universal goniometer (16, 17). There is no clear consensus on the normal ranges of IGT. However, recently most authors have used an ankle dorsiflexion index (ADI) - the difference between passive ankle dorsiflexion in standing with the knee extended vs. flexed - with a cut-off value of either 10° or 13° (7, 9, 18, 19).

The aim of this study was to determine the prevalence and extent of IGT in patients with knee or hip pathology just prior to their elective surgery and to confirm the correlations between IGT and patients' demographics, activity levels and corresponding joint pain levels.

## 2 Materials and methods

The study was approved by the National Medical Ethics Committee (approval no. 0120-169/2019/4) and has been conducted in accordance with the principles set forth in the Helsinki Declaration as revised in 2024. The patients have freely given their consent to participate in the research study and to have their data published in a journal article.

This cross-sectional study included 80 consecutive patients admitted for elective surgical procedures at the Department of Orthopaedic Surgery in the second half of 2019 due to chronic symptoms of the hip (labral tear or end-stage osteoarthritis) or knee joint (meniscus tear or end-stage osteoarthritis). Depending on the underlying pathology, patients were scheduled for either arthroscopy or arthroplasty. Each of the four therapeutic subgroups was completed once 20 participants had been successfully enrolled, ensuring a balanced distribution across the study cohort.

Exclusion criteria were applied to minimize potential confounding factors. These included the presence of neuromuscular disease, a history of previous surgical procedures distal to the knee joint, and any current or prior symptoms related to the foot or ankle region, defined as a Foot and Ankle Outcome Score below 90. For all enrolled participants, demographic data were collected, including age, sex, and body mass index (BMI). In addition, each patient's level of pain was assessed using the Visual Analogue Scale (VAS), and their level of physical activity was evaluated using the Tegner Activity Scale (TAS), as previously described (20).

Patients were divided into four subgroups, each consisting of 20 individuals, based on the type of joint pathology and age. The total knee arthroplasty (TKA) group included patients between 50 and 70 years of age with a primary diagnosis of knee osteoarthritis. The K-ARTRO group consisted of patients aged 20 to 40 years with a primary diagnosis of meniscal injury of the knee, scheduled for knee arthroscopy. The total hip arthroplasty (THA) group included patients between 50 and 70 years of age with hip osteoarthritis. Finally, the H-ARTRO group comprised patients between 20 and 40 years of age diagnosed with a labral injury of the hip and scheduled for hip arthroscopy. This structured classification enabled direct comparisons between younger and older populations, as well as between degenerative and non-degenerative joint conditions.

### 2.1 Isolated gastrocnemius tightness measurement

The IGT was assessed using the adapted WBLT, as previously described by Krause et al. (14). All measurements were performed on the day of hospital admission, prior to the elective surgical procedure. To avoid measurement errors, assessments were conducted simultaneously by two experienced musculoskeletal physiotherapists (coauthors SK and TTZ). Each patient underwent two successive measurements. In cases of discrepancy between the two measurements, additional assessments were performed until two consecutive WBLT measurements yielded identical values; this criterion was defined as a stable result. The right leg was measured from the patient's right side, and vice versa. The control leg was measured first. The patient stood barefoot, facing the wall, feet hip-width apart and aligned perpendicular to the wall. The examiner placed a gravity fluid inclinometer (Baseline Bubble Inclinometer, Fabrication Enterprises Inc., White Plains, NY, USA) on the tuber calcanei of the tested leg. The patient was instructed to slowly lunge forward, attempting to touch the knee to wall while keeping the heel of the tested leg fully in contact with the ground and the foot aligned straight ahead until the maximal dorsiflexion position was reached without heel lift. Ankle dorsiflexion was first measured with the knee flexed and then repeated with the knee fully extended. The difference in dorsiflexion between the flexed and the extended knee was used to calculate the ankle dorsiflexion index (ADI). Two cut-off points for IGT ADI ≥ 10° and ADI ≥ 13° were analysed separately, as they appear most often in the literature. However, there is no clear consensus on the appropriateness of the definition of an IGT (1). The WBLT evaluates the combined effect of the gastrocnemius on ankle dorsiflexion and does not isolate the individual contributions of the medial and lateral heads, making it unsuitable for assessing differences in their biomechanics (9).

### 2.2 Data management and statistical analysis

Data are presented as mean ± standard deviation. First, the numerical variables were tested for normality of distribution (Shapiro–Wilk test and histogram) and homogeneity of variances (Leven's test). The distribution by sex and the comparison between the groups according to the presence of IGT were checked with the chi-square test or Fisher's exact test. Depending on the distribution of the data, either parametric one-way ANOVA or non-parametric Kruskal–Wallis tests were used to compare the numerical averages between the subgroups. Based on the established hypotheses, subsequent tests for pairwise comparisons were then conducted to detect statistically significant differences between the groups. ANOVA was followed by the Games-Howell post-test or Tukey HSD (Honestly Significant Difference), while the Kruskal–Wallis test was followed by the Mann–Whitney U test. The relationship between IGT and variables such as age, sex, BMI, pain (VAS) and TAS was also analysed using the Mann–Whitney U test. Additionally, a multiple linear regression model was used to determine the strength of the relationship between these variables and IGT. The statistical significance level was set at *P* < 0.05. Statistical analysis was performed using IBM SPSS 21 (SPSS Inc., Chicago, Illinois, USA) and Microsoft Excel 2016 (Microsoft Corporation, Seattle, Washington, USA).

The sample size of 80 participants, divided into four equal subgroups of 20, was determined based on feasibility considerations and informed by prior similar studies investigating musculoskeletal characteristics in clinical populations. This number was considered sufficient to detect moderate effect sizes (Cohen's d ≈ 0.6–0.8) with a power of approximately 0.70–0.80 at an alpha level of 0.05 in between-group comparisons.

## 3 Results

The final study cohort consisted of 80 patients, comprising 44 men and 36 women, all of whom presented without any clinical symptoms in the foot or ankle region. The functional status of the foot and ankle was evaluated using the Foot and Ankle Outcome Score, with the group achieving average score of 99 ± 1, indicating preserved function and absence of symptomatic pathology in these regions. A comprehensive overview of the patients' demographic and clinical characteristics is presented in Table 1.

**Table 1:** Demographics, activity, and pain level of the participants.

Patient group (*n* = 20)	Age (years)	Gender (M/F)	BMI (kg/m^2^)	TAS (0–10)	VAS Pain (0–10)
H-ARTRO	32 ± 6	7/13	23 ± 3	3 ± 1	4 ± 2
THA	63 ± 5*	10/10	28 ± 4*	2 ± 1*	5 ± 2
K-ARTRO	34 ± 6	17/3*	24 ± 3	4 ± 1	2 ± 2*
TKA	64 ± 5*	10/10	33 ± 6**	3 ± 1*	6 ± 1

*p < 0.05 toward non-marked subgroups; **p < 0.05 toward non-marked subgroups and toward * subgroup; BMI, body mass index; TAS, Tegner Activity Scale; VAS, Visual Analogue Scale; H-ARTRO, hip arthroscopy; THA, total hip arthroplasty; K-ARTRO, knee arthroscopy; TKA, total knee arthroplasty.

The detailed results of ankle dorsiflexion measurements performed with both the knee extended and flexed, along with the corresponding ADI values, as well as the number of patients in whom IGT was identified based on both established cut-off thresholds, are presented in Table 2. For the entire study cohort, the mean ADI was calculated to be 8°, with a standard deviation of ± 4°, indicating a moderate degree of interindividual variability within the population assessed.

**Table 2:** Isolated gastrocnemius tightness (IGT) measured by the WBLT.

Limb	Patient group (*n* = 20)	DF knee extended average	DF knee flexed average	Average ADI	IGT ≥ 13°	IGT ≥ 10°
Treated limb	H-ARTRO	22˚ ± 5˚	29˚ ± 6˚	7˚ ± 4˚	2	5
	THA	18˚ ± 4˚	29˚ ± 6˚*	11˚ ± 5˚*	7*	13*
	K-ARTRO	19˚ ± 5˚	25˚ ± 4˚	6˚ ± 4˚	2	4
	TKA	17˚ ± 6˚	24˚ ± 7˚**	7˚ ± 4˚	1	7
Control	H-ARTRO	22˚ ± 6˚	29˚ ± 6˚	7˚ ± 4˚	3	6
	THA	19˚ ± 6˚	29˚ ± 6˚	10˚ ± 3˚*	5	14*
	K-ARTRO	21˚ ± 4˚	27˚ ± 4˚	6˚ ± 3˚	0	4
	TKA	18˚ ± 4˚	25˚ ± 5˚	7˚ ± 3˚	3	6

*p < 0.05 toward non-marked subgroups; **p < 0.05 toward non-marked subgroups and toward * subgroup; DF, dorsiflexion; H-ARTRO, hip arthroscopy; THA, total hip arthroplasty; K-ARTRO, knee arthroscopy; TKA, total knee arthroplasty; ADI, ankle dorsiflexion index; IGT, isolated gastrocnemius tightness.

Furthermore, multiple linear regression analysis (as presented in Table 3) revealed that age, reported pain intensity, physical activity level, and BMI were all significant predictors of the ADI across the entire patient sample. The model accounted for approximately 17.5% of the variance in ADI values (R^2^ = 0.175; *p* < 0.0001), highlighting the multifactorial nature of increased internal joint torsion and its association with both demographic and clinical variables.

**Table 3:** Regression model for across all the patients enrolled in the study.

Variable	Unstandardized coefficients		Standardized coefficients		
	B	S. E.	Beta	t	*p*
	8.801				
(constant)		1.944		4.527	0.000*
Age	0.049	0.024	0.204	2.020	0.043*
BMI	-0.100	0.062	-0.145	-1.605	0.111
TAS	-0.565	0.242	-0.210	-2.335	0.021*
VAS	0.249	0.124	0.171	2.020	0.045*

SE, standard error; *p < 0.05; BMI, body mass index; TAS, Tegner Activity Scale; VAS, Visual Analogue Scale.

## 4 Discussion

The present cross-sectional study investigated the presence and characteristics of increased IGT in patients diagnosed with common orthopaedic hip and knee pathologies prior to undergoing surgical intervention. The principal findings can be summarized as follows: IGT, defined as ADI equal to or greater than 13°, was identified in approximately 20% of patients with hip pathology and in roughly 10% of those with knee pathology. Among the various patient groups included in the study, individuals scheduled for hip arthroplasty exhibited significantly higher ADI values compared to patients in other diagnostic categories. Furthermore, statistical analyses revealed that both increasing age and higher levels of reported pain were positively associated with greater degrees of IGT. In contrast, a higher level of physical activity appeared to have a protective effect, correlating with lower ADI values and a reduced likelihood of presenting with IGT.

Several previous studies have investigated normative values of the ADI in healthy individuals by comparing the range of ankle dorsiflexion with the knee in extended vs. flexed positions. This methodological approach allows for an indirect assessment of internal joint torsion and has become a standard tool in related biomechanical research. One such study by Malhotra et al. (7) evaluated a cohort of 291 healthy adult participants and reported a mean ADI of 6°, with a standard deviation of ±3°, indicating relatively low variability within the healthy population. These findings are consistent with those reported by Chan et al. (18), who found ADI values ranging between 5.3° ± 3.4° and 6.7° ± 3.8°, depending on the subgroup analysed. Moreover, Chan et al. (18) observed a statistically significant positive correlation between ADI and increasing age (r = 0.132; *p* < 0.001), suggesting that internal joint torsion may progressively increase with aging. Conversely, a higher level of physical activity was found to be inversely correlated with ADI (r = –0.88; *p* = 0.015), supporting the notion that an active lifestyle may play a protective role in maintaining normal torsional mechanics. In the same study, an elevated ADI of 10.3° was documented in patients presenting with forefoot pathologies, further highlighting the clinical relevance of ADI as a potential marker for biomechanical deviations associated with specific musculoskeletal conditions.

Previous studies have indicated that dorsiflexion values obtained in a weight-bearing position tend to be, on average, approximately 10° higher than those measured in the supine (non-weight-bearing) position, due to the functional engagement of multiple kinetic chain components during weight-bearing assessment (10, 13). In the present study, we observed that within the subgroup of patients with hip pathology, the prevalence of IGT, defined by an ADI of ≥ 10°, approached 50%, whereas the prevalence at the more conservative cut-off of ADI ≥ 13° was approximately 20%. In contrast, among patients with knee pathology, the prevalence of IGT was notably lower, amounting to approximately 25% at ADI ≥ 10° and just over 7% at ADI ≥ 13%.

Regression analysis further revealed that the degree of ADI increased with advancing age and higher reported pain levels, while greater levels of physical activity were associated with lower ADI values. These findings may be attributable to age- and activity-related adaptations in the viscoelastic properties of the musculotendinous units, particularly those of the gastrocnemius muscle-tendon complex (21). Interestingly, body mass index did not demonstrate a statistically significant correlation with ADI in our cohort, suggesting that mechanical loading related to body weight alone may not be a decisive factor in the development of IGT. These findings are consistent with those reported in previous studies on healthy populations (7, 18, 22).

The distribution of ADI values in our patient population approximated a normal curve, with a mean value of 8° ± 4°, which is higher than the mean ADI of 6° ± 4° typically reported in healthy individuals (7, 18). This suggests a shift toward increased internal joint torsion in patients with hip and knee pathologies, which may be influenced by underlying age, pain intensity, and physical activity levels. Notably, the average ADI observed in our cohort was comparable to that reported in patients with established foot and ankle pathology (8° ± 6°), further supporting the clinical relevance of IGT beyond the distal lower limb.

We hypothesized that ADI values would vary significantly between different age groups with similar joint pathology. This assumption was confirmed in the comparison between the two hip pathology subgroups, where significant differences in ADI were found. However, this pattern did not hold true for the knee pathology subgroups, where no significant difference in ADI was observed between the younger and older groups. These findings suggest that age-related differences in IGT may be more pronounced in patients with hip pathology compared to those with knee pathology.

Moreover, the results hint at a possible association between hip joint range of motion and the presence of IGT. Existing literature supports the notion that gastrocnemius tightness may be linked to alterations in three-dimensional hip joint angles and moments during gait (23), underscoring the interconnected nature of lower limb biomechanics. It has been proposed that IGT, especially when accompanied by hamstring tightness, could influence not only the knee joint but also the function of the hip. Shortening of the gastrocnemius muscle may, therefore, affect the biomechanics of the entire posterior kinetic chain of the lower limb, including the hamstrings, hip joint, and even the lower back, contributing to compensatory movement patterns and potentially to the development or exacerbation of musculoskeletal dysfunction (5).

In this study, increased IGT was evaluated using a reliable and functionally oriented method for measuring ankle dorsiflexion. This approach is not only appropriate for research applications but also practical and easily implementable in everyday clinical settings, allowing for the efficient assessment of patients without the need for specialized equipment. We believe that the insights gained from this investigation will meaningfully contribute to the objectification and quantification of IGT in individuals presenting with hip and knee pathologies, thereby enhancing diagnostic precision. Looking forward, it would be of particular interest to identify the specific threshold of torsional tightness at which patients begin to exhibit clinical symptoms. Additionally, further research is warranted to explore the extent and nature of the correlation between IGT and various musculoskeletal pathologies affecting the hip and knee, which could provide valuable guidance for both preventative strategies and targeted therapeutic interventions.

Several limitations that may apply to this cross-sectional study must be acknowledged. All patients were assessed on the day of their hospital admission, and measurements were performed at approximately the same time point to minimize the potential influence of daily physical activity on the results of the WBLT. However, it is important to note that the precise intensity and nature of calf muscle activity in the days preceding the examination were not objectively recorded, introducing a degree of variability that could affect the measurement of ankle dorsiflexion. Patient history—including prior conservative treatments and interventions related to the index joint pathology—was obtained solely through anamnesis, which may be subject to recall bias.

An effort was made to include only individuals with a clearly defined, single-joint pathology and without clinical symptoms originating from the foot or ankle region. Nevertheless, particularly within the older subgroups undergoing total knee arthroplasty and total hip arthroplasty, it is difficult to entirely exclude the presence of coexisting conditions such as mild lumbar degenerative disease or contralateral joint involvement, both of which could potentially influence myofascial flexibility and confound the interpretation of IGT.

Given these considerations, we recommend that future studies on IGT adopt a population-based design to allow for more robust generalizability of findings, especially considering the suspected influence of age-related differences. Furthermore, the development and application of a standardized, reproducible assessment protocol would be essential to establish clinically relevant normative thresholds for dorsiflexion range of motion, both with the knee extended and flexed. Such work would enhance the diagnostic value of IGT assessment and allow for a more precise determination of its prevalence and clinical relevance across various musculoskeletal pathologies.

## 5 Conclusions

To conclude, the findings of this study indicate that increased IGT, defined by ADI equal to or exceeding 13°, was present in approximately one out of every five patients with hip pathology, and in about one out of every ten patients with knee pathology prior to surgical intervention. Since both thresholds are used in the literature, in current study ADI ≥ 13° was treated as the primary (more conservative) definition, and ADI ≥ 10° was presented as a secondary/comparative threshold. Among all the diagnostic groups evaluated, patients scheduled to undergo total hip arthroplasty demonstrated significantly higher ADI values compared to their counterparts. Furthermore, the data suggest that advancing age and higher self-reported levels of pain were positively associated with greater degrees of IGT, highlighting these as potential risk factors. In contrast, a higher level of physical activity was inversely associated with IGT, implying a potentially protective effect of regular activity on torsional joint mechanics.

## Data Availability

The raw data supporting the conclusions of this article will be made available by the authors, without undue reservation.
